# Validation of the four-miRNA biomarker panel *MiCaP* for prediction of long-term prostate cancer outcome

**DOI:** 10.1038/s41598-020-67320-y

**Published:** 2020-07-01

**Authors:** Siri H. Strand, Linnéa Schmidt, Simone Weiss, Michael Borre, Helle Kristensen, Anne Karin Ildor Rasmussen, Tina Fuglsang Daugaard, Gitte Kristensen, Hein Vincent Stroomberg, Martin Andreas Røder, Klaus Brasso, Peter Mouritzen, Karina Dalsgaard Sørensen

**Affiliations:** 1 Department of Molecular Medicine (MOMA), Aarhus University Hospital, Aarhus, Denmark; 2 Department of Clinical Medicine, Aarhus University, Aarhus, Denmark; 3 Department of Urology, Aarhus University Hospital, Aarhus, Denmark; 4 Exiqon – a Qiagen Company, Vedbæk, Denmark; 5 Department of Biomedicine, Aarhus University Hospital, Aarhus, Denmark; 6 Department of Urology, Rigshospitalet, Faculty of Health and Medical Sciences, Copenhagen Prostate Cancer Center (CPC), University of Copenhagen, Copenhagen, Denmark

**Keywords:** miRNAs, Prognostic markers, Prostate cancer

## Abstract

Improved prostate cancer prognostic biomarkers are urgently needed. We previously identified the four-miRNA prognostic biomarker panel *MiCaP* ((miR-23a-3p × miR-10b-5p)/(miR-133a-3p × miR-374b-5p)) for prediction of biochemical recurrence (BCR) after radical prostatectomy (RP). Here, we identified an optimal numerical cut-off for *MiCaP* dichotomisation using a training cohort of 475 RP patients and tested this in an independent cohort of 281 RP patients (PCA281). Kaplan–Meier, uni- and multivariate Cox regression analyses were conducted for multiple endpoints: BCR, metastatic-(mPC) and castration-resistant prostate cancer (CRPC), prostate cancer-specific (PCSS) and overall survival (OS). Functional effects of the four *MiCaP* miRNAs were assessed by overexpression and inhibition experiments in prostate cancer cell lines. We found the numerical value 5.709 optimal for *MiCaP* dichotomisation. This was independently validated in PCA281, where a high *MiCaP* score significantly [and independent of the Cancer of the Prostate Risk Assessment Postsurgical (CAPRA-S) score] predicted BCR, progression to mPC and CRPC, and PCSS, but not OS. Harrell’s C-index increased upon addition of *MiCaP* to CAPRA-S for all endpoints. Inhibition of miR-23a-3p and miR-10b-5p, and overexpression of miR-133a-3p and miR-374b-5p significantly reduced cell survival. Our results may promote future implementation of a *MiCaP-*based test for improved prostate cancer risk stratification.

## Introduction

Prostate cancer is a significant healthcare problem, globally causing > 300,000 deaths/year^1^. While many prostate cancers are indolent, a subset progress to metastatic (mPC) and castration-resistant (CRPC) disease, causing significant morbidity and mortality. Routine prognostic tools for early-stage prostate cancer are suboptimal, causing overtreatment of indolent prostate cancer and undertreatment of aggressive prostate cancer^2^. Thus, novel prognostic biomarkers are urgently needed to improve risk stratification and guide individualised treatment.

MicroRNAs (miRNAs) are small noncoding RNAs that bind complementary sequences in target messenger RNAs (mRNAs), inhibiting mRNA translation and stability^3^. miRNAs regulate genes involved in key cellular processes, including differentiation, cell-cycle control, and migration. Furthermore, dysregulation of miRNA expression is a hallmark of cancer development and progression^3,4^, and miRNAs have shown promising prognostic biomarker potential in prostate cancer^5–9^.

We recently identified the four-miRNA prognostic model *MiCaP* ((miR-23a-3p × miR-10b-5p)/(miR-133a-3p × miR-374b-5p))^9^ as an independent predictor of biochemical recurrence (BCR) in radical prostatectomy (RP) patients^9^. Here, to promote future clinical implementation of a *MiCaP* test, we identified an optimal numerical cut-off value for *MiCaP* dichotomisation using a merged training cohort of 475 RP patients (PCA475) from our previous study^9^. Next, using this cut-off, we tested and validated the prognostic potential of *MiCaP* in a novel independent cohort of 281 RP patients (PCA281).

## Results

### Establishing a numerical cut-off for patient risk stratification by *MiCaP* score

While our previous study^9^ used a fraction-based *MiCaP* score for patient stratification, we here set out to define an exact cut-off value to ease future test result interpretation.

**Figure 1:**
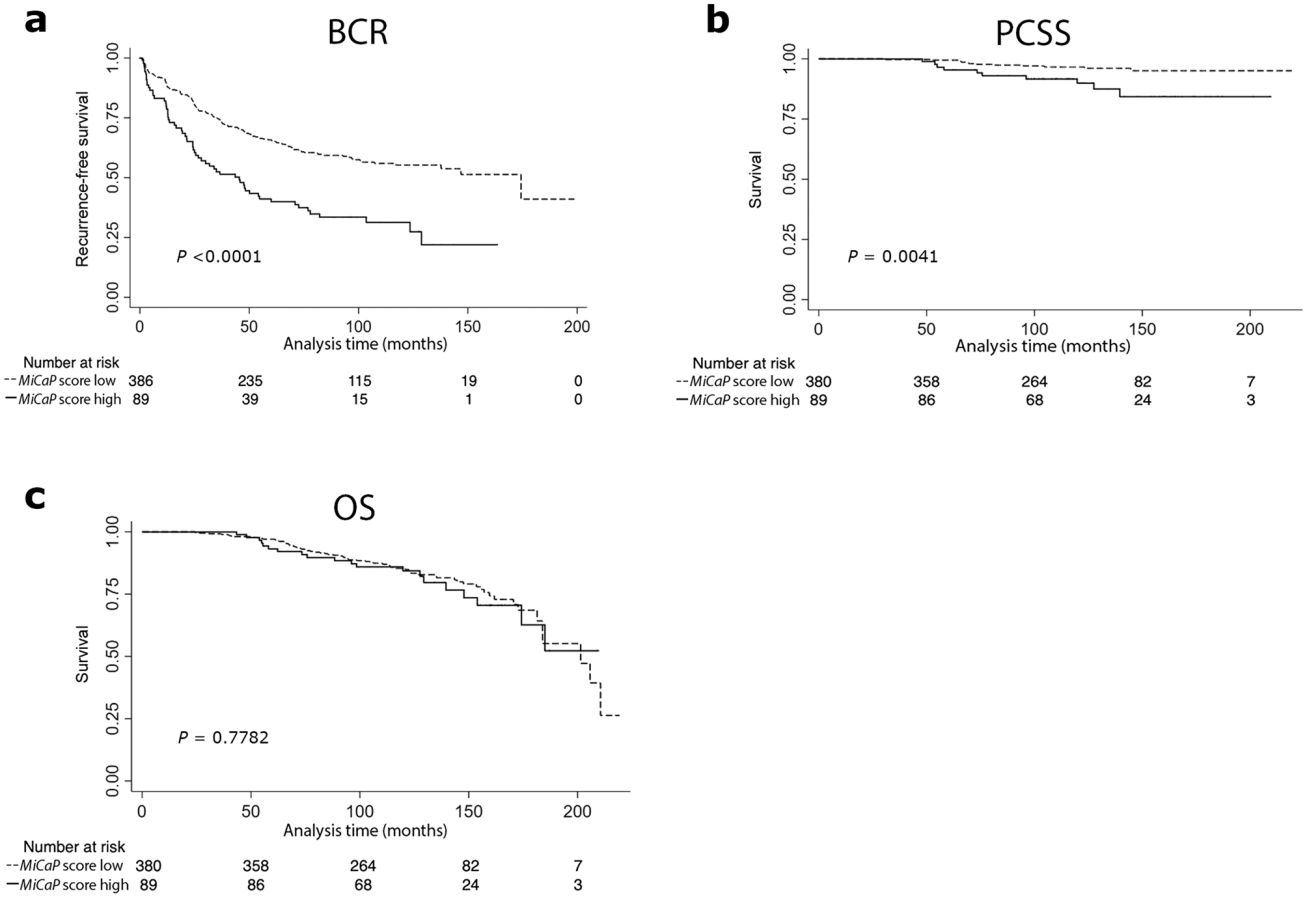
Kaplan–Meier analyses in PCA475. Kaplan–Meier analysis of patients stratified by *MiCaP* score (low vs. high) relative to three different end-points [(**a**) biochemical recurrence (BCR), (**b**) prostate cancer-specific survival (PCSS), and (**c**) overall survival (OS)]. *p* values from log-rank test.

By ROC curve analysis of BCR status at 36 months in PCA475, we identified a *MiCaP* score = 5.709 as the optimal cut-off for dichotomisation, as this value maximized both sensitivity and specificity (largest area under the curve). In this cohort, a high *MiCaP* score (≥ 5.709) was a significant predictor of BCR in Kaplan–Meier (*p* < 0.0001, Fig. 1a) and univariate Cox regression analysis (*p* < 0.0001, Table 1A). *MiCaP* remained a significant predictor of BCR also after adjusting for the well-established clinical nomogram, Cancer of the Prostate Risk Assessment Postsurgical (CAPRA-S) score (*p* = 0.002, Table 1B). Moreover, the predictive accuracy (C-index) increased from 0.702 to 0.718, when *MiCaP* was combined with CAPRA-S (Table 1B). Similar results were obtained when *MiCaP* was analysed as a continuous variable in uni- and multivariate Cox regression (*p* < 0.001, Supplementary Table S1).

**Table 1:** Cox regression analyses of *MiCaP* in both study cohorts.

A				
PCA475	HR (95% CI)	*p*	C-index	
**BCR (n = 475, 218 events)**				
*MiCaP* (high vs. low)	2.12 (1.58–2.85)	** < 0.0001**	0.564	
CAPRA-S (low vs. intermed.)	2.44 (1.63–3.65)	** < 0.0001**	0.702	
CAPRA-S (low vs. high)	8.18 (5.44–12.3)	** < 0.0001**		
**PCSS (n = 469, 23 events)**				
*MiCaP* (high vs. low)	3.14 (1.38–7.16)	**0.007**	0.613	
CAPRA-S (low vs. intermed.)	2.71 (0.58–12.8)	0.207	0.705	
CAPRA-S (low vs. high)	8.43 (1.90–37.4)	**0.005**		
**OS (n = 469, 91 events)**				
*MiCaP* (high vs. low)	1.07 (0.65–1.77)	0.778	0.511	
CAPRA-S (low vs. intermed.)	1.57 (0.89–2.78)	0.121	0.604	
CAPRA-S (low vs. high)	2.67 (1.51–4.71)	**0.001**		

B				
PCA475	HR (95% CI)	*p*	C-index	C-index
**BCR (n = 475, 218 events)**				
*MiCaP* (high vs. low)	1.63 (1.21–2.20)	**0.002**		0.718
CAPRA-S (low vs. intermed.)	2.41 (1.61–3.60)	** < 0.0001**	0.702	
CAPRA-S (low vs. high)	7.54 (4.99–11.4)	** < 0.0001**		
**PCSS (n = 469, 23 events)**				
*MiCaP* (high vs. low)	2.44 (1.05–5.65)	**0.037**		0.735
CAPRA-S (low vs. intermed.)	2.64 (0.56–12.4)	0.219	0.705	
CAPRA-S (low vs. high)	7.14 (1.59–32.0)	**0.01**		

C				
PCA281	HR (95% CI)	*p*	C-index	
**BCR (n = 281, 121 events)**				
*MiCaP* (high vs. low)	1.80 (1.21–2.68)	**0.004**	0.563	
CAPRA-S (low vs. intermed.)	2.40 (1.38–4.17)	**0.002**	0.692	
CAPRA-S (low vs. high)	6.75 (3.89–11.7)	** < 0.001**		
**mPC (n = 281, 35 events)**				
*MiCaP* (high vs. low)	3.77 (1.92–7.40)	** < 0.001**	0.655	
CAPRA-S (low vs. intermed.)	1.92 (0.59–6.18)	0.276	0.724	
CAPRA-S (low vs. high)	8.16 (2.71–24.5)	** < 0.001**		
**CRPC (n = 281, 24 events)**				
*MiCaP* (high vs. low)	3.22 (1.44–7.20)	**0.004**	0.659	
CAPRA-S (low vs. intermed.)	2.13 (0.43–10.6)	0.354	0.748	
CAPRA-S (low vs. high)	10.1 (2.32–43.8)	**0.002**		
**PCSS (n = 281, 14 events)**				
*MiCaP* (high vs. low)	5.06 (1.76–14.6)	**0.003**	0.694	
CAPRA-S (low vs. intermed.)	0.69 (0.10–4.93)	0.716	0.738	
CAPRA-S (low vs. high)	6.28 (1.37–28.7)	**0.018**		
**OS (n = 281, 57 events)**				
*MiCaP* (high vs. low)	1.47 (0.82–2.62)	0.192	0.544	
CAPRA-S (low vs. intermed.)	1.66 (0.80–3.48)	0.176	0.61	
CAPRA-S (low vs. high)	2.87 (1.37–6.04)	**0.005**		

D				
PCA281	HR (95% CI)	*p*	C-index	C-index
**BCR (n = 281, 121 events)**				
*MiCaP* (high vs. low)	1.57 (1.05–2.35)	**0.026**		0.701
CAPRA-S (low vs. intermed.)	2.31 (1.33–4.02)	**0.003**	0.692	
CAPRA-S (low vs. high)	6.47 (3.72–11.2)	** < 0.001**		
**mPC (n = 281, 35 events)**				
*MiCaP* (high vs. low)	2.88 (1.45–5.71)	**0.002**		0.785
CAPRA-S (low vs. intermed.)	1.75 (0.54–5.66)	0.352	0.724	
CAPRA-S (low vs. high)	6.60 (2.16–20.1)	**0.001**		
**CRPC (n = 281, 24 events)**				
*MiCaP* (high vs. low)	2.38 (1.06–5.38)	**0.037**		0.806
CAPRA-S (low vs. intermed.)	1.99 (0.40–9.87)	0.401	0.748	
CAPRA-S (low vs. high)	8.45 (1.92–37.2)	**0.005**		
**PCSS (n = 281, 14 events)**				
*MiCaP* (high vs. low)	3.75 (1.28–11.0)	**0.016**		0.807
CAPRA-S (low vs. intermed.)	0.62 (0.09–4.44)	0.638	0.738	
CAPRA-S (low vs. high)	4.73 (1.01–22.2)	**0.048**		

Univariate (A, C) and multivariate (B, D) Cox regression analysis of *MiCaP* (analysed as a dichotomised variable) and CAPRA-S (low, intermediate, high) relative to three different end-points [biochemical recurrence (BCR), prostate cancer-specific survival (PCSS), and overall survival (OS)] in PCA475 (A, B) and five different end-points (BCR, metastatic prostate cancer (mPC), castration-resistant prostate cancer (CRPC), PCSS, and OS) in PCA281 (C, D). *p* values < 0.05 in bold. Multivariate analysis was not carried out relative to OS in either cohort, as statistical significance was not reached in univariate analysis.

Furthermore, a high (vs. low) *MiCaP* score was significantly associated with poor prostate cancer-specific survival (PCSS) in PCA475, as assessed by Kaplan–Meier (*p* = 0.0041, Fig. 1b) and univariate Cox regression analysis (*p* = 0.007, Table 1A). After adjusting for CAPRA-S, *MiCaP* remained a significant independent predictor of PCSS in PCA475 (*p* = 0.037, Table 1B). The C-index increased from 0.705 to 0.735 when adding *MiCaP* to CAPRA-S (Table 1B). Moreover, *MiCaP* was a borderline significant predictor of PCSS when analysed as a continuous variable in univariate Cox regression (*p* = 0.072, Supplementary Table S1).

There was no significant association between *MiCaP* and overall survival (OS) in PCA475 (Fig. 1c, Table 1A, Supplementary Table S1).

### Independent validation of *MiCaP:* Biochemical recurrence-free survival

For independent validation, we used a novel cohort of 281 RP patients (PCA281). Here, CAPRA-S high-risk patients had significantly higher *MiCaP* scores than CAPRA-S low- and intermediate-risk patients (*p* = 0.003, and *p* = 0.024, respectively, Supplementary Fig. S1), supporting the link between a high *MiCaP* score and more aggressive prostate cancer also observed in PCA475 (*p* = 0.0001 and *p* = 0.011, respectively, Supplementary Fig. S1).

**Figure 2:**
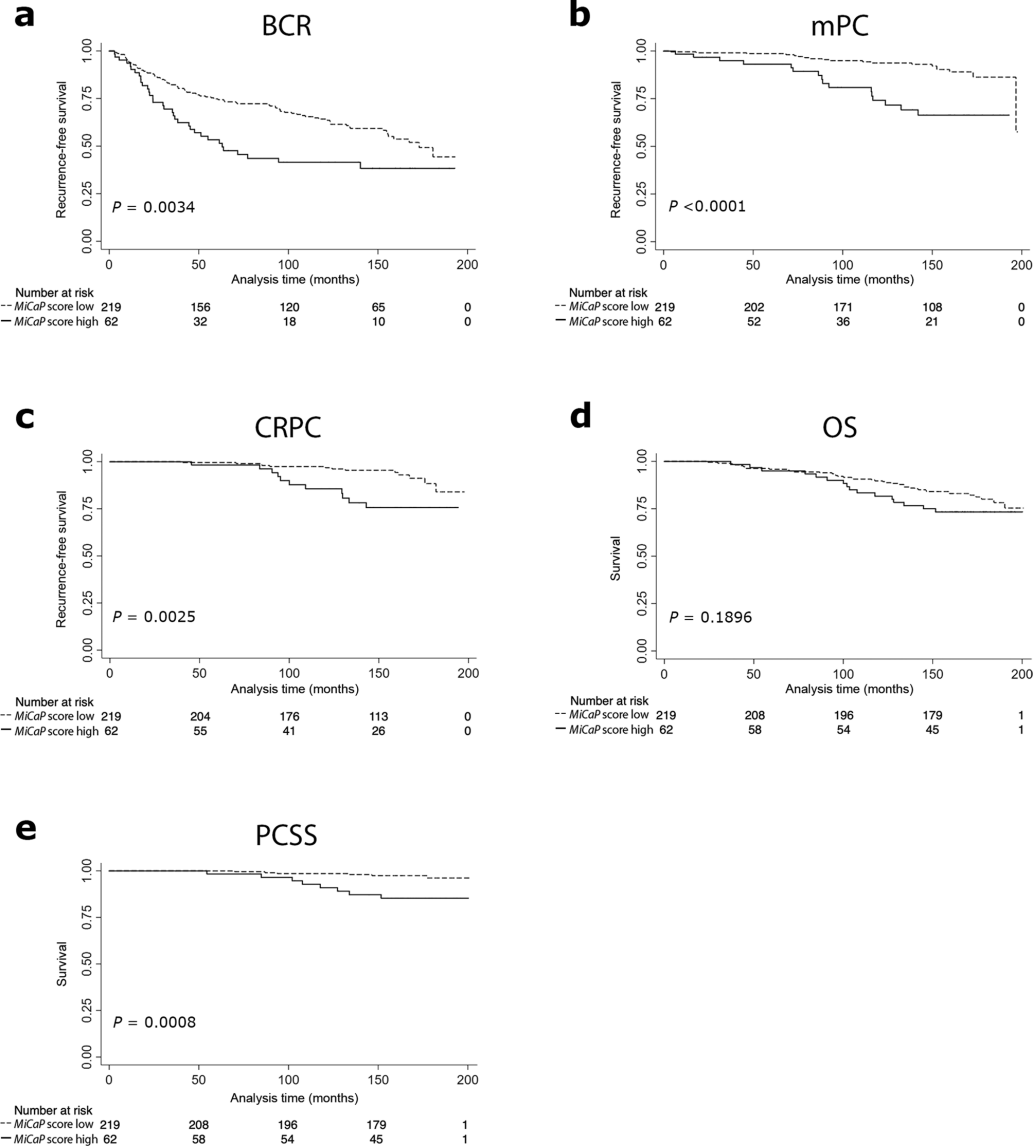
Kaplan–Meier analyses in PCA281. Kaplan–Meier analysis of patients stratified by *MiCaP* score (low vs. high) relative to five different end-points. (**a**) Biochemical recurrence (BCR), (**b**) metastatic prostate cancer (mPC), (**c**) castration-resistant prostate cancer (CRPC), (**d**) overall survival (OS), and (**e**) prostate cancer-specific survival (PCSS). *p* values from log-rank test.

Next, using the numerical *MiCaP* cut-off defined in PCA475, patients in the PCA281 validation cohort were stratified into high- or low-risk groups. In PCA281, patients with a high *MiCaP* score had significantly higher risk of BCR in both Kaplan–Meier (*p* = 0.0034, Fig. 2a) and univariate Cox regression analysis (*p* = 0.004, Table 1C). *MiCaP* remained a significant predictor of BCR after adjusting for CAPRA-S (*p* = 0.026, Table 1D), and the C-index increased from 0.692 to 0.701 when *MiCaP* was added to CAPRA-S (Table 1D). Similar results were obtained when *MiCaP* was analysed as a continuous variable in the PCA281 validation cohort (multivariate Cox regression: *p* ≤ 0.041, Supplementary Table S2).

### *MiCaP* predicts progression to metastatic and castration resistant prostate cancer

Patients in PCA281 with a high (vs. low) *MiCaP* score had significantly higher risk of progression to mPC, as assessed by Kaplan–Meier analysis (*p* < 0.0001, Fig. 2b) and univariate Cox regression analysis (*p* < 0.001, Table 1C). In multivariate analysis, a high *MiCaP* score was a significant predictor of metastatic progression independent of CAPRA-S (*p* = 0.002, Table 1D), and addition of *MiCaP* to CAPRA-S increased the C-index from 0.724 to 0.785 (Table 1D).

Moreover, PCA281 patients with a high (vs. low) *MiCaP* score had significantly higher risk of progression to CRPC, as assessed by Kaplan–Meier analysis (*p* = 0.0025, Fig. 2c). This was corroborated by uni- and multivariate Cox regression analyses, where *MiCaP* was a significant predictor of CRPC, also after adjustment for CAPRA-S (*p* = 0.004 and *p* = 0.037, respectively, Table 1C, 2D). Additionally, adding *MiCaP* to CAPRA-S increased the C-index from 0.748 to 0.806 (Table 1D). Similar results were obtained when *MiCaP* was analysed as a continuous variable relative to both mPC and CRPC (multivariate Cox regression: *p* ≤ 0.022, Supplementary Table S2).

### Overall- and prostate cancer-specific survival analyses

In PCA281, we found no significant associations between *MiCaP* and OS by Kaplan–Meier nor univariate Cox regression analyses (Fig. 2d, Table 1C, Supplementary Table S2), confirming the results from the training cohort (Fig. 1c, Table 1A, Supplementary Table S1).

In PCA281, patients with a high (vs. low) *MiCaP* score showed significantly shorter PCSS by Kaplan–Meier (*p* = 0.0008, Fig. 2e) and univariate Cox regression analysis (*p* = 0.003, Table 1C). Furthermore, a high *MiCaP* score remained a significant adverse predictor of PCSS after adjusting for CAPRA-S (*p* = 0.016, Table 1D), and addition of *MiCaP* to CAPRA-S improved the C-index from 0.738 to 0.807 (Table 1D). Comparable results were obtained when *MiCaP* was analysed as a continuous variable relative to PCSS (multivariate Cox regression: *p* = 0.005, Supplementary Table S2).

### Assessment of progressed patients by *MiCaP* score

We next investigated the fraction of patients progressed by *MiCaP* score. Here, patients were ranked by *MiCaP* score and assigned to one of three groups (top 33%: high, middle 33%: intermediate, and bottom 33%: low). The number of patients progressed in each group was calculated for all relevant endpoints in both cohorts. In PCA475, patients in the high *MiCaP* score group had the highest number of events for both BCR [60.1% vs. 44.7% (intermediate) and 32.9% (low)] and PCSS (high score: 7.6%, intermediate: 3.1%, low: 3.8%) (Supplementary Fig. S3). These results were validated by analysis in PCA281 for both BCR (high: 51.1%, intermediate: 40.9%, low: 37.2%) and PCSS (high: 10.6%, intermediate: 2.2%, low: 2.1%, Supplementary Fig. S3). Moreover, 21.3% in the high *MiCaP* score group in PCA281 progressed to mPC, compared to 8.6% (intermediate) and 7.4% (low). Finally, 16% in the high *MiCaP* score group progressed to CRPC, compared to 5.4% in the intermediate and 4.3% in the low risk groups (Supplementary Fig. S3). These results show that the risk of recurrence, progression, or cancer-specific death increases with the *MiCaP* score, further strengthening *MiCaP* as a highly clinically relevant biomarker candidate.

### Functional assessment of miRNAs in prostate cancer cell lines

**Figure 3:**
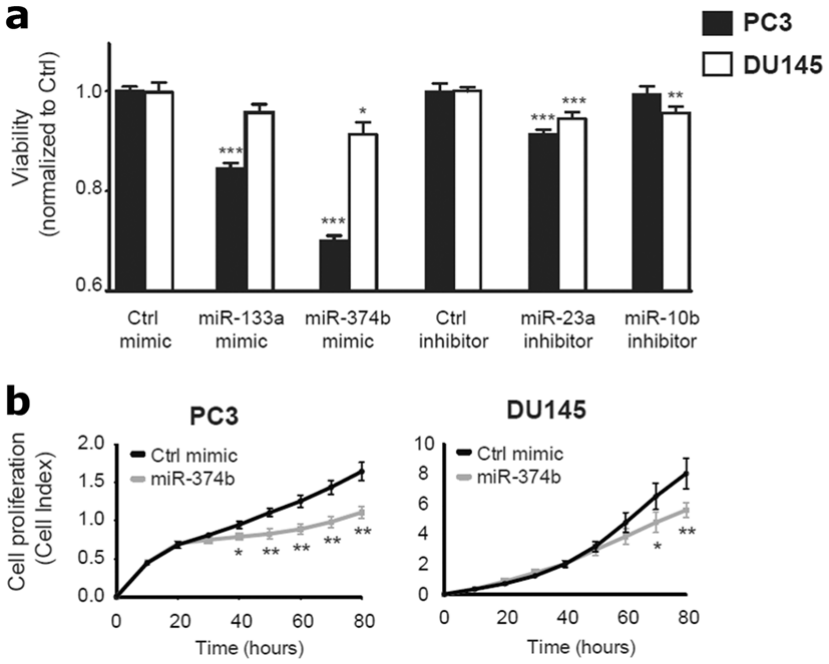
Overexpression and inhibition studies of miRNAs. Functional studies of miRNAs in prostate cancer cell lines. (**a**) Inhibitory effect on prostate cancer cell viability by single miRNA mimics and inhibitors in PC3 and DU145. Each mimic or inhibitor was compared to the corresponding control mimic or inhibitor in the same cell line. Results from alamarBlue viability assay (72 h post-transfection), plotted as mean ± SE of three independent experiments performed in triplicate. (**b**) Significant inhibitory effect on real-time proliferation by miR-374b-5p mimic transfections in PC3 and DU145 using the xCELLigence instrument. Results from one representative experiment performed in triplicate (three experiments in total) are plotted as mean ± SD for each time point. Student’s two-sided *t*-test, *p < 0.05, **p < 0.01, ***p < 0.001.

To explore the functional effects on prostate cancer cell survival of the miRNAs included in the *MiCaP* model, we transfected PC3 and DU145 cells with either mimics of miR-133a-3p or miR-374b-5p, or inhibitors of miR-23a-3p or miR-10b-5p. Overexpression of miR-133a-3p or miR-374b-5p significantly reduced PC3 cell viability (*p* < 0.001, Fig. 3a), indicating a tumour suppressor role for these miRNAs in prostate cancer. A similar trend was observed in DU145 cells but was significant only for miR-374b-5p (*p* = 0.034, Fig. 3a). Conversely, inhibition of miR-23a-3p caused a moderate, statistically significant reduction in PC3 and DU145 cell viability (*p* < 0.001, Fig. 3a), while inhibition of miR-10b-5p slightly reduced cell viability in DU145 (*p* = 0.001, Fig. 3a), but not in PC3 (*p* = 0.613, Fig. 3a).

To further investigate miR-374b-5p, for which no previous functional studies in prostate cancer cells have been reported, we assessed real-time cell proliferation. Overexpression of miR-374b-5p significantly inhibited proliferation of PC3 and DU145 cells (*p* = 0.003 and *p* = 0.006 at 80 h, respectively, Fig. 3b).

In summary, each of the four *MiCaP* miRNAs significantly affected prostate cancer cell survival in at least one of the cell lines investigated, consistent with their direction of deregulation in aggressive prostate cancer.

## Discussion

We recently identified the promising four-miRNA prognostic ratio model *MiCaP* for prediction of BCR and PCSS after RP^9^. Thus, *MiCaP* may help identify RP patients with a high risk of adverse outcome, who therefore may need adjuvant therapy or intensified post-RP follow-up. First, to facilitate future clinical implementation, we trained an optimal numerical cut-off value for *MiCaP* using 475 RP patients analysed previously^9^. Next, using this cut-off, we confirmed the independent prognostic potential of *MiCaP* in a novel cohort (PCA281). This is the first report to demonstrate a significant association between *MiCaP* and risk of progression to mPC/CRPC, and to show *MiCaP* as an independent adverse prognostic factor for PCSS in two distinct prostate cancer patient cohorts. Furthermore, our functional studies demonstrated tumour suppressor roles for miR-133a-3p and miR-374b-5p and oncogenic roles for miR-23a-3p and miR-10b-5p in prostate cancer cells, providing a likely biological basis for the link between a high *MiCaP* score in prostate cancer tumours and a more aggressive disease course.

Previous prostate cancer biomarker discovery studies have proposed multi-miRNA prognostic panels^10–12^, but these require additional normalisation. This is circumvented by using a ratio model such as *MiCaP*. Likewise, our training and validation of an exact cut-off should also ease future clinical translation. Prior to *MiCaP*, only one study had explored the prognostic potential of a miRNA-based ratio model for prostate cancer, but lacked multivariate analysis and analysed only 145 patients^13^. In contrast, we have tested *MiCaP* in four cohorts (> 1,200 patients), including our previous study^9^, demonstrating its robustness and independent prognostic value beyond the CAPRA-S nomogram.

Addition of parameters, such as novel biomarkers, to existing prognostic models or clinical nomograms often results in minor C-index increases^14^, thus raising concerns about the added clinical value upon inclusion of the parameters. However, when *MiCaP* was added to the CAPRA-S nomogram, notable C-index increases were observed for all endpoints, at levels comparable to results for commercially available prognostic gene expression signatures such as Decipher^15^, Prolaris^16^, and Oncotype^17^. This indicates that *MiCaP* may be used in addition to standard clinicopathological assessment to improve the accuracy of prostate cancer patient risk stratification. Further studies are warranted in order to investigate if *MiCaP* can predict metastatic disease progression specifically after salvage radiotherapy at BCR, as has been previously reported for the Decipher test^18^.

We found that high expression levels of miR-10b-5p and miR-23a-3p were associated with adverse outcome, consistent with an oncogenic role for these miRNAs in prostate cancer. Similarly, elevated pre-miR-10b expression in prostate cancer has previously been associated with poor recurrence-free survival^19^. Elevated miR-10b-5p expression has also been associated with shorter survival in glioma^20^ and non-small-cell lung cancer^21^, whereas miR-10b-5p has been reported as downregulated in breast and renal cancer^22,23^. In seeming contrast to our findings, two previous studies reported that miR-23a-3p was downregulated in prostate cancer vs. normal tissue^24,25^. However, each of these studies investigated no more than 20 patients, possibly explaining this discrepancy. Moreover, downregulation of miR-23a-3p has been associated with high clinical stage and worse survival in oral squamous cell carcinoma^26^ and melanoma^27^, while the opposite was found for miR-23a-3p in renal cell carcinoma^28^, indicating that the regulation of miR-10b-5p and miR-23a-3p is tissue type-specific.

miR-133a-3p has previously been reported as downregulated in prostate cancer^29^ and in other cancer types, including breast^30^, gastric^31^, oesophageal^32^, and colon cancer^33^, suggesting a tumour suppressor function of this miRNA across these cancer types.

In line with our results^9^, one earlier study found miR-374b-5p to be downregulated in prostate cancer vs. normal tissue^34^. Similarly, low expression of miR-374b-5p has been associated with worse outcome in breast cancer^35^ and miR-374b-5p has been reported to be downregulated also in pancreatic^36^, ovarian^37^, and bladder cancer^38^. Conversely, in head and neck cancer, high expression of miR-374b-5p has been correlated with worse prognosis^39^, together indicating a tissue-type specific role for miR-374b-5p.

Altogether, these findings highlight the need for thorough individual assessment of the four miRNAs, as their roles in cancer seem to be diverse and are not yet fully elucidated.

Here, we assessed the effect of miR-23a-3p on prostate cancer cell viability for the first time and found that miR-23a-3p inhibition decreased DU145 and PC3 cell viability. Consistent with this oncogenic role, Wen et al.^40^ showed that miR-23a-3p overexpression stimulated DU145 cell invasion. In contrast to our findings, Cai et al*.*^24^ reported that miR-23a-3p overexpression inhibited PC3 and DU145 invasion and migration, although they did not investigate the effect on cell viability specifically.

As another novel finding, we showed that inhibition of miR-10b-5p reduced DU145 cell viability, indicating an oncogenic role. Consistent with this, a previous study showed that pre-miR-10b overexpression promoted DU145 cell migration^19^. In contrast, Tang et al*.* showed that miR-10b-5p inhibited proliferation and migration of prostate cancer cells, although they did not specify in which cell lines^41^.

Additionally, we showed that miR-133a-3p overexpression decreased PC3 cell viability*.* In support of this tumour suppressor function, previous studies reported miR-133a-3p overexpression to increase apoptosis^29^ and decrease viability, migration, and invasion^42^ of prostate cancer cells.

Finally, this is the first study to investigate the functional role of miR-374b-5p in prostate cancer cells. We demonstrated a tumour suppressor function for miR-374b-5p in prostate cancer, as overexpression significantly decreased PC3 and DU145 cell viability and proliferation. Overexpression of miR-374b-5p has been shown to decrease migration and invasion of bladder cancer cell lines^38^ and viability^37^ of ovarian cancer cell lines, in agreement with a tumour suppressor role for miR-374b-5p.

The target genes and molecular pathways mediating the potential oncogenic or tumour suppressor effects observed here for the four *MiCaP* miRNAs remain to be elucidated, but this is considered to be beyond the scope of the present work. However, it has been previously reported that miR-23a-3p interacts directly with PAK6, hereby regulating the cell cytoskeleton via LIMK1 and cofilin^24^. Cytoskeletal changes are required for metastasis^43^, thus providing a possible link with tumour aggressiveness, although this requires further investigation. Overexpression of miR-10b-5p has been shown to inhibit HAS3 expression, a hyaluronan synthase that can inhibit tumour growth^44^. This mechanism of action for miR-10b-5p is in line with our results, which demonstrated an oncogenic role of miR-10b-5p in prostate cancer. Overexpression of miR-133a-3p has been reported to downregulate EGFR^42^, a receptor tyrosine kinase, which is known to play a role in the development of androgen-independent prostate cancer^45^. Several of the downstream EGFR effectors were also inactivated upon miR-133a-3p overexpression, including phosphorylated ERK and AKT and MMP-2. The latter is an EGFR effector mediating cell migration and invasion. Thus, it is possible that the tumour suppressive effects of miR-133a-3p on viability, migration, and invasion in prostate cancer cells may be caused by an interaction with EGFR^42^. Finally, no previous studies have characterized the targets of miR-374b-5p in prostate cancer cells. In bladder cancer, however, overexpression of miR-374b-5p has been shown to repress ZEB2^38^. ZEB2 is a master regulator of epithelial-mesenchymal transition, an important first step in metastasis^46^; these results are therefore in line with the tumour suppressive effect of miR-374b-5p also reported in our study.

An interesting observation from several previous reports is that miR-23a-3p^24,40^, miR-10b-5p^19,41^, and miR-133a-3p^42^ all seem to be involved in regulation of prostate cancer cell invasion/migration in vitro. Corroborating these earlier findings, our results showed that *MiCaP* could predict mPC (Fig. 2b). It would thus be interesting to further explore the link between the *MiCaP* miRNAs and metastasis in the future. Such future studies should also help identify key target genes for these miRNAs in prostate cancer cells.

There are some potential limitations to this study. Only RP patients were investigated. Hence, the prognostic potential of *MiCaP* in other prostate cancer patient groups remains to be investigated. Moreover, the analyses were based on RP specimens. Future studies should analyse prostate cancer tissue samples from diagnostic needle biopsies to examine the potential of *MiCaP* as a pre-operative prognostic biomarker. Implementation of MR-guided biopsies^47^ should reduce sampling errors, and thus likely increase the clinical value of tissue-based molecular tests such as *MiCaP*. Future studies should also examine the prognostic potential of *MiCaP* in minimally-invasive liquid biopsies^9^. Furthermore, we observed a relatively high rate of positive margin in PCA281. It must be emphasized that PCA281 was a historical cohort of unscreened men who underwent open retropubic RP in 2002–2005 with a median tumour volume of 12.8 ml^48^, which is twice the volume of a modern cohort of high-risk prostate cancer patients undergoing RP in Europe^49^. It is well known that tumour parameters are the most important risk factors for positive surgical margins following RP^50^.

Some potential limitations also exist regarding the cell line experiments. First, we did not investigate which target genes directly mediated the phenotypic effects of the four miRNAs in prostate cancer cells. Second, we only tested androgen-independent prostate cancer cell lines. Thus, to fully elucidate the role of the four miRNAs in prostate cancer development and progression, future studies should include a broader panel of prostate cancer cell lines, including also androgen-sensitive cell lines. Third, future functional studies should include also cell migration and invasion experiments, in order to investigate in more detail the molecular mechanisms that link a high *MiCaP* score with increased risk of progression to mPC. However, additional cell line studies are considered to be beyond the scope of the present study, the main focus of which was the independent clinical validation of *MiCaP*.

In conclusion, this study established an optimal numerical cut-off value for *MiCaP* testing and validated *MiCaP* as a significant independent predictor of mPC and CRPC, in addition to BCR and PCSS, in a novel independent cohort of 281 RP patients. Furthermore, we present the first functional studies demonstrating a tumour suppressor role of miR-374b-5p in prostate cancer cells. Future studies should examine the prognostic potential of *MiCaP* in diagnostic needle biopsies and liquid biopsies to assess if *MiCaP* can improve risk stratification at time of diagnosis.

## Materials and methods

### Ethics statement

All research was carried out in accordance with relevant guidelines and regulations. Written informed consent was obtained from all participants. The studies were approved by The Central Denmark Region Committees on Health Research Ethics [#2000/0,299 (PCA475)], the Danish National Committee on Health Research Ethics [#H-6–2014-111 (PCA281)], and The Danish Data Protection Agency [#2013–41-2041 (PCA475) and #2006–41-6,256 (PCA281)]. Follow-up was updated in April 2018 (PCA475) and October 2018 (PCA281).

### Patients

PCA475: This cohort consisted of 475 RP patients (Table 2) from the combined PCA123 and PCA352 cohorts described previously^9^ (inclusion/exclusion criteria: Supplementary Fig. S2). Briefly, tumour tissue samples from RP patients were collected at the Department of Urology, Aarhus University Hospital, Aarhus, Denmark between 1997 and 2005.

**Table 2:** Clinical and histopathological variables of the study cohorts.

	PCA475	PCA281
**Samples**	RP (*N* = 475)	RP (*N* = 281)
**Median age, years (IQR)**	63.8 (59.9–67.5)	62.5 (59.2–66.5)
**Median preOP PSA [ng/mL] (IQR)**	11.4 (8.20–17.5)	10.0 (6.80–14.0)
**Pathologic T-stage**		
pT2a-c	314 (66%)	189 (67%)
pT3a	113 (24%)	53 (19%)
pT3b	44 (9%)	39 (14%)
Unknown	4 (1%)	0
**Gleason grade group**		
Grade I (GS < 7)	138 (29%)	115 (41%)
Grade II (GS = 3 + 4)	193 (41%)	103 (37%)
Grade III (GS = 4 + 3)	67 (14%)	40 (14%)
Grade IV (GS = 8)	66 (14%)	12 (4%)
Grade V (GS > 8)	10 (2%)	11 (4%)
Unknown	1 (0.2%)	0
**Surgical margin status**		
Negative	335 (71%)	118 (42%)
Positive	140 (29%)	163 (58%)
**Biochemical recurrence status**		
No reccurence	257 (54%)	160 (57%)
Reccurence	218 (46%)	121 (43%)
**mPC status**		
No metastases	Not available	246 (88%)
Metastases	Not available	35 (13%)
**CRPC status**		
No CRPC	Not available	257 (92%)
CRPC	Not available	24 (9%)
**CAPRA-S**		
Low	140 (29%)	81 (29%)
Intermediate	213 (45%)	125 (45%)
High	113 (24%)	75 (27%)
Unknown	9 (2%)	0
**Median follow-up time, months (IQR)**	117.9 (91.0–143.0)	152.1 (113.2–167.7)
**Survival status**		
Alive	378 (80%)	224 (80%)
Dead	91 (19%)	57 (20%)
Prostate cancer-specific deaths	23 (5%)	14 (5%)
Unknown	6 (1%)	0

Clinicopathological characteristics of patients in PCA475 (training cohort) and PCA281 (validation cohort).

PCA281: Prostate cancer tissue samples were collected from 314 RP patients between 2002 and 2005 at the Department of Urology, Rigshospitalet, Copenhagen, Denmark^48^. After exclusion of 33 patients (Supplementary Fig. S2), the final cohort consisted of 281 patients (Table 2).

In all cases, androgen deprivation therapy was protocolised according to clinical guidelines in Denmark. Furthermore, all prostatectomy samples were re-graded according to the ISUP 2005 Gleason grading system^51^ and reported in accordance with ISUP 2014 Gleason grade group criteria^52^.

### RNA extraction and RT-qPCR

Total RNA was extracted from archived (FFPE) prostatectomy samples (Supplementary Table S3), using the Qiagen (Hilden, Germany) miRNeasy FFPE kit. MicroRNA expression was quantified using the miRCURY LNA™ Universal RT microRNA PCR platform (Exiqon, Vedbæk, Denmark)^5,7,9^. Briefly, 50 ng RNA was reverse transcribed in 10 µl reactions using the miRCURY LNA™ Universal RT microRNA PCR, Polyadenylation and cDNA synthesis kit (Exiqon). Next, cDNA was diluted 100 × for RT-qPCR, and miRNA expression levels analysed on the microRNA Ready-to-Use PCR platform (Exiqon) in 384-well plates with ExiLENT SYBR Green master mix (Qiagen). Amplification reactions were run on a LightCycler 480 Real-Time PCR System (Roche, Basel, Switzerland) and analysed using the Roche LC software^9^.

### Statistical analyses

For each patient, the *MiCaP* score was calculated from raw Cq values according to logarithmic rules: *MiCaP* = (Cq_miR-133a-3p_ + Cq_miR-374b-5p_)—(Cq_miR-23a-3p_ + Cq_miR-10b-5p_)^9^. Statistical analyses were performed using STATA v.15.0 (StataCorp, College Station, Texas, USA). The STATA code is included in the Supplementary Information. Furthermore, a TRIPOD checklist is included as Supplementary Fig. S4. *p *values < 0.05 were considered significant. Associations between *MiCaP* score and CAPRA-S were assessed using Wilcoxon rank-sum tests. CAPRA-S risk groups were defined as previously reported^53^: Low-risk (CAPRA-S ≤ 2), intermediate-risk (CAPRA-S = 3–5), high-risk (CAPRA-S ≥ 6). The prognostic potential of *MiCaP* was analysed by uni/multivariate Cox regression analyses, Kaplan–Meier analyses, and log-rank tests. Predictive accuracy was determined using Harrell’s concordance index (C-index)^54^. For analyses of *MiCaP* as a dichotomised variable (high vs. low), the cut-off was determined by ROC curve analysis of BCR status at 36 months in PCA475, as this value maximized both sensitivity and specificity (largest area under the curve). This cut-off (*MiCaP* = 5.709) was used for stratification in both PCA475 and PCA281. Clinical endpoints in survival analyses were (1) BCR, defined as prostate specific antigen (PSA) ≥ 0.2 ng/ml; (2) Progression to mPC, defined by medical journal entry; (3) Progression to CRPC, defined by castration level serum testosterone (< 1.7 nmol/l) in combination with either biochemical progression (PSA increase > 50% in two measurements) or radiological progression (≥ 2 new lesions); (4) OS; and (5) PCSS. For BCR-free survival, patients who did not experience BCR were censored at their last normal PSA measurement. For mPC- or CRPC-free survival analyses, patients who did not experience metastasis or CRPC were censored at the date of last follow-up or death. For OS and PCSS analyses, living patients were censored at the date of survival data extraction from the Danish Civil Registration System. For functional studies, statistical analyses were conducted in GraphPad Prism (GraphPad 6.0, La Jolla, California, USA). Student’s two-sided *t*-test was used to assess differences between groups.

### Cell culture and transfections

PC3 (RRID:CVCL_0035) and DU145 (RRID:CVCL_0105) prostate cancer cell lines were obtained from the American Type Culture Collection and cultured in RPMI medium (Lonza, Basel, Switzerland) supplemented with 10% fetal bovine serum and 1% penicillin/streptomycin. Cells were validated as Mycoplasma-free using the MycoSensor PCR Assay kit (Cat#302,108, Stratagene, La Jolla, California, USA), and cultured in antibiotics-free medium 24 h prior to transfection. Authenticity of cell lines was verified by short tandem repeat analysis (identicell.dk) within 3 years prior to the experiments. All cell line experiments were performed within a maximum of three months culturing after thawing of individual cells stocks (aliquots). MicroRNA mimic and inhibitor transfections were performed by a reverse protocol using Lipofectamine 2000 (Thermo Fisher Scientific, Waltham, Massachusetts, USA). Cells were transfected with mirVana miR-23a-3p inhibitor (Product ID: MH10644), mirVana miR-10b-5p inhibitor (Product ID: MH11108), mirVana miR-133a-3p mimic (Product ID: MC10413), mirVana miR-374b-5p mimic (Product ID: MC11339) or relevant negative controls (mirVana miRNA Mimic, Negative Control #1, Cat#4,464,058, and mirVana miRNA Inhibitor, Negative Control #1, Cat#4,464,076) (Thermo Fisher Scientific). Before initiating functional experiments, transfection efficiencies were assessed using a Cy3-labeled pre-miR Negative Control (catalog number AM17120; Ambion, Applied Biosystems, Waltham, MA) and was near to 100% at 48 h after transfection for both PC3 and DU145. Negative controls were used for normalisation.

### Viability and proliferation assays

Cells were seeded at 6,000 (PC3) or 5,000 (DU145) cells/well in 96-well plates at the time of transfection. Cell viability was assessed 72 h post transfection using alamarBlue (Thermo Fisher Scientific). Fluorescence was recorded using a Synergy HT-reader (BioTek, Winooski, Vermont, USA). Cell proliferation was analysed in 16-well plates on the xCELLigence Real-Time Cell Analyzer (RTCA, Roche). Experiments were performed in triplicates and repeated at least three times.

## Supplementary information

Supplementary file1 (PDF 1028 kb)

## Data Availability

Data available on request from the authors.
