# Supplementary material for: Validation of the four-miRNA biomarker panel MiCaP for prediction of long-term prostate cancer outcome
Source: Sci Rep. 2020 Jul 1;10:10704. doi: 10.1038/s41598-020-67320-y (PMC7329825; doi:10.1038/s41598-020-67320-y)
Supplement: Supplementary file 1 — Supplementary file1 (PDF 1028 kb) [file 41598_2020_67320_MOESM1_ESM.pdf]

Supplementary Information

Validation of the four-miRNA biomarker panel *MiCaP* for prediction of long-term prostate cancer outcome

Siri H. Strand[1,2], Linnéa Schmidt[1,2], Simone Weiss[1,2], Michael Borre[2,3], Helle Kristensen[4], Anne Karin Ildor Rasmussen[4], Tina Fuglsang Daugaard[5], Gitte Kristensen[6], Hein Vincent Stroomberg[6], Martin Andreas Røder[6], Klaus Brasso[6], Peter Mouritzen[4], Karina Dalsgaard Sørensen[1,2*]

[1]Department of Molecular Medicine (MOMA), Aarhus University Hospital, Aarhus, Denmark
[2]Department of Clinical Medicine, Aarhus University, Aarhus, Denmark
[3]Department of Urology, Aarhus University Hospital, Aarhus, Denmark
[4]Exiqon – a Qiagen company, Vedbæk, Denmark
[5]Department of Biomedicine, Aarhus University Hospital, Aarhus, Denmark
[6]Department of Urology, Rigshospitalet, Faculty of Health and Medical Sciences, Copenhagen Prostate Cancer Center (CPC), University of Copenhagen, Copenhagen, Denmark
[*]Corresponding author. E-mail: kdso@clin.au.dk

Supplementary Figure S1. Distribution of *MiCaP* scores according to CAPRA-S risk groups.

Boxplot of *MiCaP* score in patients from PCA281 (A, validation cohort) and PCA475 (B, training cohort), stratified by CAPRA-S risk groups (low, intermediate, high). *P*-values from Wilcoxon rank-sum test. * = p<0.05.

A

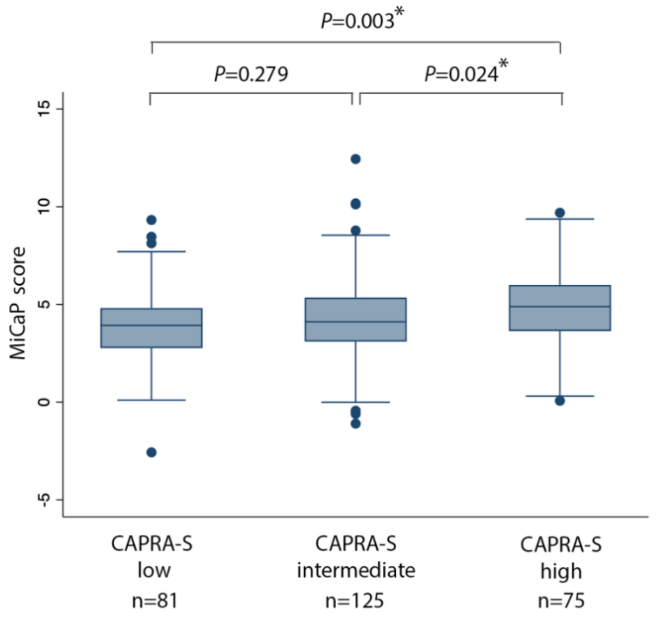

B

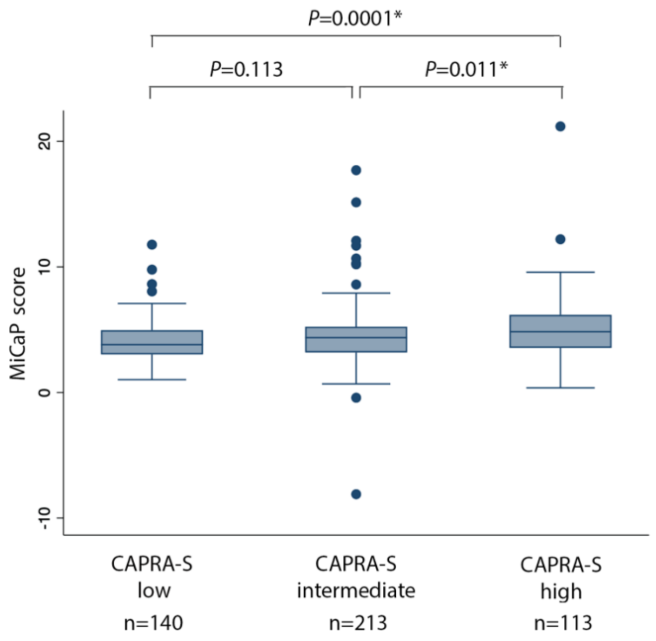

Supplementary Figure S2. Inclusion of samples in PCA475 and PCA281.

Sample inclusion of PCA475 (A) and PCA281 (B) according to REMARK[1] guidelines.

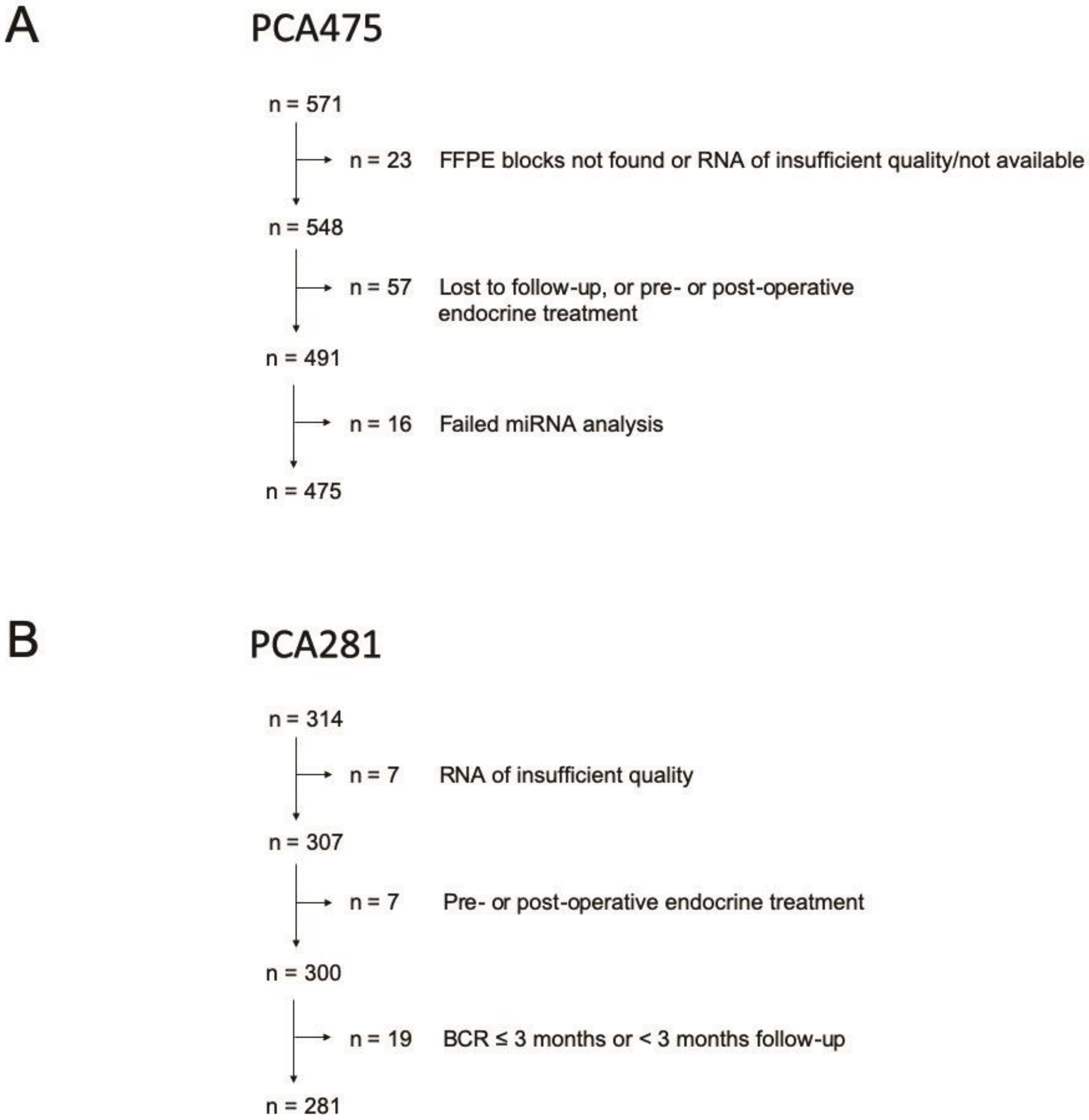

Supplementary Figure S3. Assessment of progressed patients by *MiCaP* score.

Percentage progressed patients by *MiCaP* score. Patients in both cohorts were ranked by *MiCaP* score and assigned into one of three groups based on this ranking (high: top 33%, intermediate: middle 33%, and low: bottom 33%). The number of patients progressed was calculated for each available endpoint in each group in each cohort (PCA475: BCR, biochemical recurrence. PCSS, prostate cancer-specific survival. PCA281: BCR, biochemical recurrence. mPC, metastatic prostate cancer. CRPC, castration resistant prostate cancer. PCSS, prostate cancer specific survival).

A

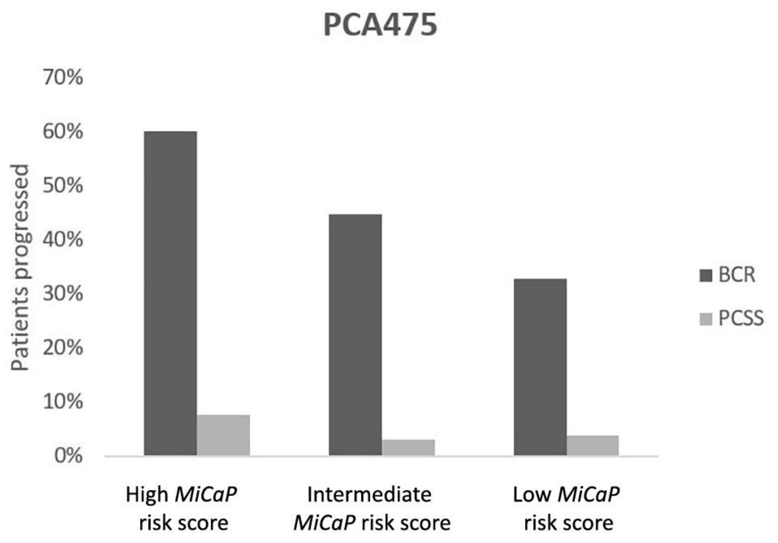

B

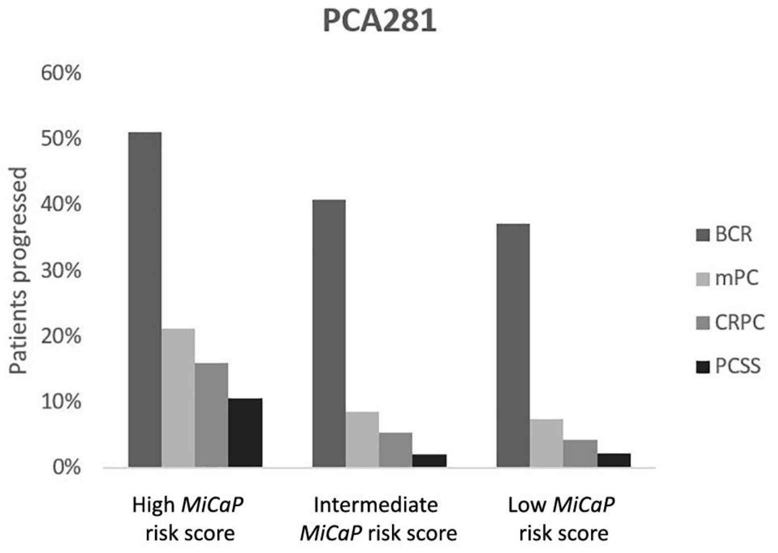

Supplementary Figure S4. TRIPOD checklist.

TRIPOD Checklist: Prediction Model Development

Section/Topic	Item	Checklist Item	Page
**Title and abstract**			
Title	1	Identify the study as developing and/or validating a multivariable prediction model, the target population, and the outcome to be predicted.	1
Abstract	2	Provide a summary of objectives, study design, setting, participants, sample size, predictors, outcome, statistical analysis, results, and conclusions.	1
**Introduction**			
Background and objectives	3a	Explain the medical context (including whether diagnostic or prognostic) and rationale for developing or validating the multivariable prediction model, including references to existing models.	1
	3b	Specify the objectives, including whether the study describes the development or validation of the model or both.	1
**Methods**			
Source of data	4a	Describe the study design or source of data (e.g., randomized trial, cohort, or registry data), separately for the development and validation data sets, if applicable.	8
	4b	Specify the key study dates, including start of accrual; end of accrual; and, if applicable, end of follow-up.	8
Participants	5a	Specify key elements of the study setting (e.g., primary care, secondary care, general population) including number and location of centres.	8
	5b	Describe eligibility criteria for participants.	8 & Suppl. Fig. S2
	5c	Give details of treatments received, if relevant.	8
Outcome	6a	Clearly define the outcome that is predicted by the prediction model, including how and when assessed.	8
	6b	Report any actions to blind assessment of the outcome to be predicted.	Not relevant
Predictors	7a	Clearly define all predictors used in developing or validating the multivariable prediction model, including how and when they were measured.	8
	7b	Report any actions to blind assessment of predictors for the outcome and other predictors.	Not relevant
Sample size	8	Explain how the study size was arrived at.	Suppl. Fig. S2
Missing data	9	Describe how missing data were handled (e.g., complete-case analysis, single imputation, multiple imputation) with details of any imputation method.	No imputation
Statistical analysis methods	10a	Describe how predictors were handled in the analyses.	8
	10b	Specify type of model, all model-building procedures (including any predictor selection), and method for internal validation.	8
	10d	Specify all measures used to assess model performance and, if relevant, to compare multiple models.	8
Risk groups	11	Provide details on how risk groups were created, if done.	8
**Results**			
Participants	13a	Describe the flow of participants through the study, including the number of participants with and without the outcome and, if applicable, a summary of the follow-up time. A diagram may be helpful.	8, Table 1 & suppl. Fig. S3
	13b	Describe the characteristics of the participants (basic demographics, clinical features, available predictors), including the number of participants with missing data for predictors and outcome.	Table 2
Model development	14a	Specify the number of participants and outcome events in each analysis.	Table 1, fig. 1-2
	14b	If done, report the unadjusted association between each candidate predictor and outcome.	Table 1A, 1C
Model specification	15a	Present the full prediction model to allow predictions for individuals (i.e., all regression coefficients, and model intercept or baseline survival at a given time point).	8
	15b	Explain how to the use the prediction model.	8
Model performance	16	Report performance measures (with CIs) for the prediction model.	Table 1, fig.1-2
**Discussion**			
Limitations	18	Discuss any limitations of the study (such as nonrepresentative sample, few events per predictor, missing data).	7-8
Interpretation	19b	Give an overall interpretation of the results, considering objectives, limitations, and results from similar studies, and other relevant evidence.	8
Implications	20	Discuss the potential clinical use of the model and implications for future research.	8
**Other information**			
Supplementary information	21	Provide information about the availability of supplementary resources, such as study protocol, Web calculator, and data sets.	8, 10
Funding	22	Give the source of funding and the role of the funders for the present study.	11

Supplementary Table S1. Cox regression analyses of *MiCaP* (as a continuous variable) in PCA475.

Univariate (A) and multivariate (B) Cox regression analysis of *MiCaP* (analysed as a continuous variable) and CAPRA-S (low, intermediate, high) relative to three different end-points (biochemical recurrence (BCR), prostate cancer-specific survival (PCSS), and overall survival (OS)) in PCA475. *P*-values <0.05 in bold. Multivariate analysis was not carried out relative to PCSS or OS, as statistical significance was not reached in univariate analysis.

A

**PCA475 (Univariate analyses)**	**BCR (n=475, 218 events)**		
	**HR (95% CI)**	**P**	**C-index**
MiCaP (continuous)	1.15 (1.10 – 1.22)	**<0.0001**	0.594
CAPRA-S (low vs. intermed.)	2.44 (1.63 - 3.65)	**<0.0001**	0.702
CAPRA-S (low vs. high)	8.18 (5.44 - 12.3)	**<0.0001**	
	**PCSS (n=469, 23 events)**		
	**HR (95% CI)**	**P**	**C-index**
MiCaP (continuous)	1.12 (0.99 – 1.27)	0.072	0.598
CAPRA-S (low vs. intermed.)	2.71 (0.58 - 12.8)	0.207	0.705
CAPRA-S (low vs. high)	8.43 (1.90 - 37.4)	**0.005**	
	**OS (n=469, 91 events)**		
	**HR (95% CI)**	**P**	**C-index**
MiCaP (continuous)	1.05 (0.97 - 1.14)	0.224	0.541
CAPRA-S (low vs. intermed.)	1.57 (0.89 - 2.78)	0.121	0.604
**CAPRA-S (low vs. high)**	**2.67 (1.51 - 4.71)**	**0.001**	

B

**PCA475 (Multivariate analysis)**	**BCR (n=475, 218 events)**			
	**HR (95% CI)**	**P**	**C-index**	**C-index**
MiCaP (continuous)	1.11 (1.05 – 1.18)	**<0.001**		0.723
CAPRA-S (low vs. intermed.)	2.38 (1.59 - 3.56)	**<0.001**	0.702	
CAPRA-S (low vs. high)	7.52 (4.99 - 11.4)	**<0.001**		

Supplementary Table S2. Cox regression analyses of *MiCaP* (as a continuous variable) in PCA281.

Univariate (A) and multivariate (B) Cox regression analysis of *MiCaP* (analysed as a continuous variable) and CAPRA-S (low, intermediate, high) relative to five different end-points (BCR, metastatic prostate cancer (mPC), castration-resistant prostate cancer (CRPC), PCSS, and OS). *P*-values <0.05 in bold. Multivariate analysis was not carried out relative to OS, as statistical significance was not reached in univariate analysis.

A

**PCA281 (Univariate analyses)**	**BCR (n=281, 121 events)**		
	**HR (95% CI)**	**P**	**C-index**
MiCaP (continuous)	1.12 (1.03 - 1.22)	**0.009**	0.577
CAPRA-S (low vs. intermed.)	2.40 (1.38 - 4.17)	**0.002**	0.692
CAPRA-S (low vs. high)	6.75 (3.89 - 11.7)	**<0.001**	
	**mPC (n=281, 35 events)**		
	**HR (95% CI)**	**P**	**C-index**
MiCaP (continuous)	1.30 (1.13 - 1.49)	**<0.001**	0.682
CAPRA-S (low vs. intermed.)	1.92 (0.59 - 6.18)	0.276	0.724
CAPRA-S (low vs. high)	8.16 (2.71 - 24.5)	**<0.001**	
	**CRPC (n=281, 24 events)**		
	**HR (95% CI)**	**P**	**C-index**
MiCaP (continuous)	1.28 (1.08 - 1.51)	**0.004**	0.676
CAPRA-S (low vs. intermed.)	2.13 (0.43 - 10.6)	0.354	0.748
CAPRA-S (low vs. high)	10.1 (2.32 - 43.8)	**0.002**	
	**PCSS (n=281, 14 events)**		
	**HR (95% CI)**	**P**	**C-index**
MiCaP (continuous)	1.41 (1.15 - 1.74)	**0.001**	0.722
CAPRA-S (low vs. intermed.)	0.69 (0.10 - 4.93)	0.716	0.738
CAPRA-S (low vs. high)	6.28 (1.37 - 28.7)	**0.018**	
	**OS (n=281, 57 events)**		
	**HR (95% CI)**	**P**	**C-index**
MiCaP (continuous)	1.00 (0.87 - 1.14)	0.952	0.494
CAPRA-S (low vs. intermed.)	1.66 (0.80 - 3.48)	0.176	0.610
CAPRA-S (low vs. high)	2.87 (1.37 - 6.04)	**0.005**	

B

PCA281 (Multivariate analyses)	BCR (n=281, 121 events)			
	**HR (95% CI)**	**P**	**C-index**	**C-index**
MiCaP (continuous)	1.09 (1.02 - 1.19)	**0.041**		0.701
CAPRA-S (low vs. intermed.)	2.30 (1.33 - 4.01)	**0.003**	0.692	
CAPRA-S (low vs. high)	6.48 (3.73 - 11.3)	**<0.001**		
	**mPC (n=281, 35 events)**			
	**HR (95% CI)**	**P**	**C-index**	**C-index**
MiCaP (continuous)	1.27 (1.09 - 1.49)	**0.002**		0.784
CAPRA-S (low vs. intermed.)	1.67 (0.51 - 5.49)	0.396	0.724	
CAPRA-S (low vs. high)	6.84 (2.24 - 21.0)	**0.001**		
	**CRPC (n=281, 24 events)**			
	**HR (95% CI)**	**P**	**C-index**	**C-index**
MiCaP (continuous)	1.24 (1.03 - 1.50)	**0.022**		0.808
CAPRA-S (low vs. intermed.)	1.87 (0.37 - 9.36)	0.446	0.748	
CAPRA-S (low vs. high)	8.43 (1.92 - 37.0)	**0.005**		
	**PCSS (n=281, 14 events)**			
	**HR (95% CI)**	**P**	**C-index**	**C-index**
MiCaP (continuous)	1.43 (1.11 – 1.83)	**0.005**		0.830
CAPRA-S (low vs. intermed.)	0.54 (0.07 – 3.93)	0.541	0.738	
CAPRA-S (low vs. high)	4.63 (1.00 – 21.5)	**0.050**		

Supplementary Table S3. miRNA assays used in the study.

miRNA	Assay Cat. No (Qiagen)
hsa-miR-23a-3p	YP00204772
hsa-miR-10b-5p	YP00205637
hsa-miR-133a-3p	YP00204788
hsa-miR-374b-5p	YP00204608

STATA code for statistical analysis of clinical data.

```
//ROC analysis to identify optimal cutpoint
use "D:\miCaP\STATA\DATA\PCA475.dta", clear
set more off
roctab bcr_36_mo micap, detail

*** Box plots, Wilcoxon ***
*** PCA475 ***

graph box micap, over (t_capras) ytitle ("miCaP")
*low vs. int:
drop if t_capras==2
ranksum micap, by (t_capras)
clear
use "D:\miCaP\STATA\DATA\PCA475.dta", clear
set more off
*low vs. high:
drop if t_capras==1
ranksum micap, by (t_capras)
clear
use "D:\miCaP\STATA\DATA\PCA475.dta", clear
set more off
*int vs. high:
drop if t_capras==0
ranksum micap, by (t_capras)
clear

*** PCA281 ***
use "D:\miCaP\STATA\DATA\PCA281.dta", clear
set more off
graph box micap, over (t_capras) ytitle ("miCaP")
```

```
*low vs. int:
drop if t_capras==2
ranksum micap, by (t_capras)
clear
use "D:\miCaP\STATA\DATA\PCA281.dta", clear
set more off
*low vs. high:
drop if t_capras==1
ranksum micap, by (t_capras)
clear
use "D:\miCaP\STATA\DATA\PCA281.dta", clear
set more off
*int vs. high:
drop if t_capras==0
ranksum micap, by (t_capras)
clear

*** SURVIVAL ANALYSES ***

*** PCA475 ***

*** BCR ***
use "D:\miCaP\STATA\DATA\PCA475.dta", clear
set more off
generate d_miCaP=1 if micap >= 5.709
recode d_miCaP (mis = 0)
stset rfs_months, failure (bcr==1) scale(1)
stcox micap
estat concordance
xi:stcox micap i.t_capras
estat concordance
```

```
xi:stcox i.d_miCaP

estat concordance

xi:stcox i.t_capras

estat concordance

xi:stcox i.d_micap i.t_capras

estat concordance

sts graph, by (d_miCaP) risktable plot1opts(lpattern(-) lcolor(black)) plot2opts (lpattern(l)
lcolor(black))

sts test d_miCaP


*** PCSS ***

stset time_to_death_mo, failure (pc_death==1) scale(1)

stcox micap

estat concordance

xi:stcox micap i.t_capras

estat concordance

xi:stcox i.d_miCaP

estat concordance

xi:stcox i.t_capras

estat concordance

xi:stcox i.d_micap i.t_capras

estat concordance

sts graph, by (d_miCaP) risktable plot1opts(lpattern(-) lcolor(black)) plot2opts (lpattern(l)
lcolor(black))

sts test d_miCaP

clear


*** PCA281 ***


*** BCR ***

use "D:\miCaP\STATA\DATA\PCA281.dta", clear

set more off
```

```
generate d_miCaP=1 if micap >= 5.709

recode d_miCaP (mis = 0)

stset rfs_months, failure (bcr==1) scale(1)

stcox micap

estat concordance

xi:stcox micap i.t_capras

estat concordance

xi:stcox i.d_miCaP

estat concordance

xi:stcox i.t_capras

estat concordance

xi:stcox i.d_micap i.t_capras

estat concordance

sts graph, by (d_miCaP) risktable plot1opts(lpattern(-) lcolor(black)) plot2opts (lpattern(l) lcolor(black))

sts test d_miCaP


*** mPC ***

stset time_to_dist_meths, failure (dist_mets==1) scale(1)

stcox micap

estat concordance

xi:stcox micap i.t_capras

estat concordance

xi:stcox i.d_miCaP

estat concordance

xi:stcox i.t_capras

estat concordance

xi:stcox i.d_micap i.t_capras

estat concordance

sts graph, by (d_miCaP) risktable plot1opts(lpattern(-) lcolor(black)) plot2opts (lpattern(l) lcolor(black))

sts test d_miCaP

*** CRPC ***
```

```
stset time_to_crpc_months, failure (crpc==1) scale(1)

stcox micap

estat concordance

xi:stcox micap i.t_capras

estat concordance

xi:stcox i.d_miCaP

estat concordance

xi:stcox i.t_capras

estat concordance

xi:stcox i.d_micap i.t_capras

estat concordance

sts graph, by (d_miCaP) risktable plot1opts(lpattern(-) lcolor(black)) plot2opts (lpattern(l) lcolor(black))

sts test d_miCaP


*** PCSS ***

stset time_to_death_mo, failure (pc_death==1) scale(1)

stcox micap

estat concordance

xi:stcox micap i.t_capras

estat concordance

xi:stcox i.d_miCaP

estat concordance

xi:stcox i.t_capras

estat concordance

xi:stcox i.d_micap i.t_capras

estat concordance

sts graph, by (d_miCaP) risktable plot1opts(lpattern(-) lcolor(black)) plot2opts (lpattern(l) lcolor(black))

sts test d_miCaP

clear
```

References

1 McShane, L. M. *et al.* REporting recommendations for tumour MARKer prognostic studies (REMARK). *Eur J Cancer* **41**, 1690-1696, doi:10.1016/j.ejca.2005.03.032 (2005).
